# Optical genome mapping improves structural variant detection and characterization in syndromic and neurogenetic disorders

**DOI:** 10.1007/s00439-026-02856-z

**Published:** 2026-09-10

**Authors:** Rosa Catalina Lederbogen, Sabine Hoffjan, Cornelia Köhler, Charlotte Thiels, Ulrike Angelika Mau-Holzmann, Sylke Singer, Sabrina Schreiber, Anne Purczeld, Rebecca Buchert, Maria Viktorovna Yusenko, Hoa Huu Phuc Nguyen, Wanda Maria Gerding

**Affiliations:** 1 Department of Human Genetics, Ruhr-University, 44801 Bochum, Germany; 2 Center for Rare Diseases Ruhr (CeSER), 44791 Bochum, Germany; 3 Department of Neuropediatrics, University Children’s Hospital, Ruhr-University Bochum, 44791 Bochum, Germany; 4 Institute of Medical Genetics and Applied Genomics, University Tübingen, 72076 Tübingen, Germany; 5 MVZ Dr. Eberhard & Partner, 44137 Dortmund, Germany; 6 LADR Medizinisches Versorgungszentrum Recklinghausen eGbR, 45659 Recklinghausen, Germany

## Abstract

Optical Genome Mapping (OGM) offers superior resolution compared to standard diagnostic methods such as karyotyping and FISH, enabling the detection of nearly all types of chromosomal aberrations with non-centromeric breakpoints. This study evaluated OGM’s potential to enhance the genetic findings in unsolved cases of neurogenetic and syndromic disease requiring further investigation after standard genetic testing. In 10 patients with various neurogenetic diagnoses, OGM confirmed all structural findings previously detected by karyotyping, chromosomal microarray (CMA), and/or NGS. Moreover, OGM provided additional structural insights in five cases, such as identifying a novel candidate gene in a patient with a balanced translocation, redefining of breakpoint regions in familial translocations, characterization of complex rearrangements, and revising of initial diagnostic interpretations. Most importantly, we present OGM results for three individuals with ring chromosomes 18, 20, and 22, highlighting the need to adjust filter settings and to incorporate the rare variant pipeline for accurate detection. Based on our experiences, we propose a strategic approach for identifying ring chromosomes using OGM. On the other hand, OGM did not identify causative variants in three unsolved cases with strong clinical suspicion of hereditary neuropathy. In summary, while OGM did not yield new insights for hereditary neuropathy, it provided additional or refined information in 6 out of 10 cases with other syndromic diseases. These findings underscore the value of OGM in increasing the diagnostic yield and precision of genetic testing.

**Supplementary Information:**

The online version contains supplementary material available at https://doi.org/10.1007/s00439-026-02856-z.

## Introduction

Neurogenetic diseases encompass a wide range of conditions, including neurodevelopmental disorders, epilepsy, hereditary neuropathies, and congenital malformations (Flore and Milunsky 2012; Hickman et al. 2022). Establishing a correct diagnosis is important for affected individuals and their families because it can provide information about the possible spectrum of symptoms and life prognosis, guide reproductive decisions and enable access to social support by connecting with other affected families. However, many of these diseases are rare, and patients often experience a prolonged diagnostic odyssey before a causal diagnosis is achieved (Rosell et al. 2016). Standard diagnostic tests for unclear constitutional diseases include karyotyping, chromosomal microarray (CMA), and next-generation sequencing (NGS) (Iqbal et al. 2023). In (neuro)developmental disorders, the diagnostic yield using whole exome sequencing (WES) is 36%, followed by 15–20% using CMA and less than 5% by karyotyping (Srivastava et al. 2019; Mantere et al. 2021). In epilepsy, gene panels or exome sequencing show diagnostic yields of approximately 24% (Stefanski et al. 2021) and in hereditary neuropathies about 24–45% (Gonzaga-Jauregui et al. 2015; Hartley et al. 2017). Therefore, despite these advancements in genetic testing, many patients remain undiagnosed, stressing the need for additional emerging technologies (Wojcik et al. 2023). Long-read sequencing and optical genome mapping (OGM) have been increasingly evaluated in recent years for a range of applications (Warburton and Sebra 2023; Kernohan and Boycott 2024). OGM offers a whole-genome structural analysis based on DNA molecules from about 250 kb reaching up to the mega base range. Thus, OGM enables the detection of large structural variants (SVs) with a significantly higher resolution than karyotyping (Mantere et al. 2021; Hu et al. 2021). It detects SVs, such as deletions, insertions, and rearrangements from 500 bp on (Dremsek et al. 2021), as well as large copy number variations (CNVs), typically analyzed by CMA, by the use of two different analysis pipelines (Xu et al. 2024). Structural aberrations are known to cause various neurogenetic and syndromic diseases (Lupski 2015; Monlong et al. 2018; Dremsek et al. 2021; Barseghyan et al. 2022; Alibutud et al. 2023). OGM has already proven to be effective in diagnosing monogenic disorders unsolved by standard diagnostic methods, such as infantile epilepsy, Duchenne muscular dystrophy and neuropathy (Cope et al. 2021; Hsueh et al. 2023; Facchini et al. 2023; Schrauwen et al. 2024). Furthermore, OGM can identify complex chromosomal rearrangements that are challenging in routine diagnostic techniques (Shi et al. 2023; van der Sanden et al. 2024; Shim et al. 2024).

The present study comprises 10 patients with neurogenetic disorders, all of whom had likely causative genetic findings identified through routine diagnostics. The phenotypes include syndromic and non-syndromic developmental disorders, epilepsy, congenital malformations and one case with a familial syndromic disorder. Additionally, based on previous success of OGM in resolving neuropathy cases (Facchini et al. 2023; Rahikkala et al. 2024), three unsolved patients with a strong clinical suspicion of hereditary neuropathy were included. The study aimed at evaluating whether OGM has the potential to enhance diagnostic accuracy in comparison to prior genetic results in patients affected by syndromic and neurogenetic disorders.

## Material and methods

### Patients

A total of 13 patients, eight females and five males, were recruited at the department of Human Genetics, Ruhr-University Bochum or the University Children`s Hospital / St. Josef Hospital Bochum. 10 patients from the neurodevelopmental disorder spectrum had likely causative genetic findings (either directly or within family history) which could not be completely clarified using standard diagnostic methods (karyotyping, microarray and WES and/or short read whole genome sequencing (WGS)). Additionally, three patients with neuropathy, either presenting with childhood onset and severe disease course or with a family history suggestive of autosomal dominant inheritance were included in this study. In these patients, standard diagnostic workup (*PMP22* deletion/duplication analysis and WES) did not identify a pathogenic aberration. Detailed clinical information about the individual patients is given in Supplementary document 1, Table 5. All patients or their parents/legal guardians gave their written consent to the participation in this study and publication of the results.

EDTA-blood was collected and frozen at -40 °C until preparation. DNA preparation of ultra-high molecular weight (UHMW)-gDNA was done using the Prep SP-G2 Frozen Human Blood DNA Isolation CG-00006 kit, Rev B (Bionano Genomics 2023), following the manufacturer's instructions. UHMW-gDNA labeling followed the Bionano Prep® DLS-G2 protocol (CG-30553-1, Rev E), using the Bionano Prep DLS-G2 kit. DNA concentration was determined using a Qubit fluorometer (invitrogen Qubit Fluorometer 3, Thermo Fisher Scientific, Waltham, MA, USA). UHMW-gDNA was loaded on a Saphyr Chip G2.3 and run on a Saphyr system, where DNA molecules were linearized in nanochannels, imaged and bioinformatically processed using Bionano software solutions. Quality parameters were evaluated according to the manufacturer's guidelines (Bionano Genomics 2024).

### Data analysis

The Bionano Solve™ data analysis software (v3.7_20221013_25) was used to assemble single molecule maps into consensus maps, and identified SVs and CNVs were compared to the hg38/GRCh38 reference genome. Genome mapping was visualized using the Bionano Access software (v1.7.2). The analysis was done using the de novo pipeline, with a resolution of 500 bp for insertions, 700 bp for deletions, 30 kb for duplications and inversions and 70 kb for translocations (Bionano Genomics 2021a, b). The identified SVs were filtered with standard settings and a frequency of 1% or less compared to the Bionanogenomics control population datasets provided by the manufacturer. This control population includes 285 ethnically diverse genomes without known diseases. In some cases, additional analysis was performed using the rare variant (RV) and guided assembly pipelines, and hg38 preferred and hg38 known canonical mapped features were used for detailed analysis of exonic and intronic regions provided by the Bionanogenomics software. All aberrations identified by OGM were individually evaluated for pathogenicity using OMIM, NCBI, DECIPHER, Franklin, and PubMed databases.

## Results

**Fig. 1:**
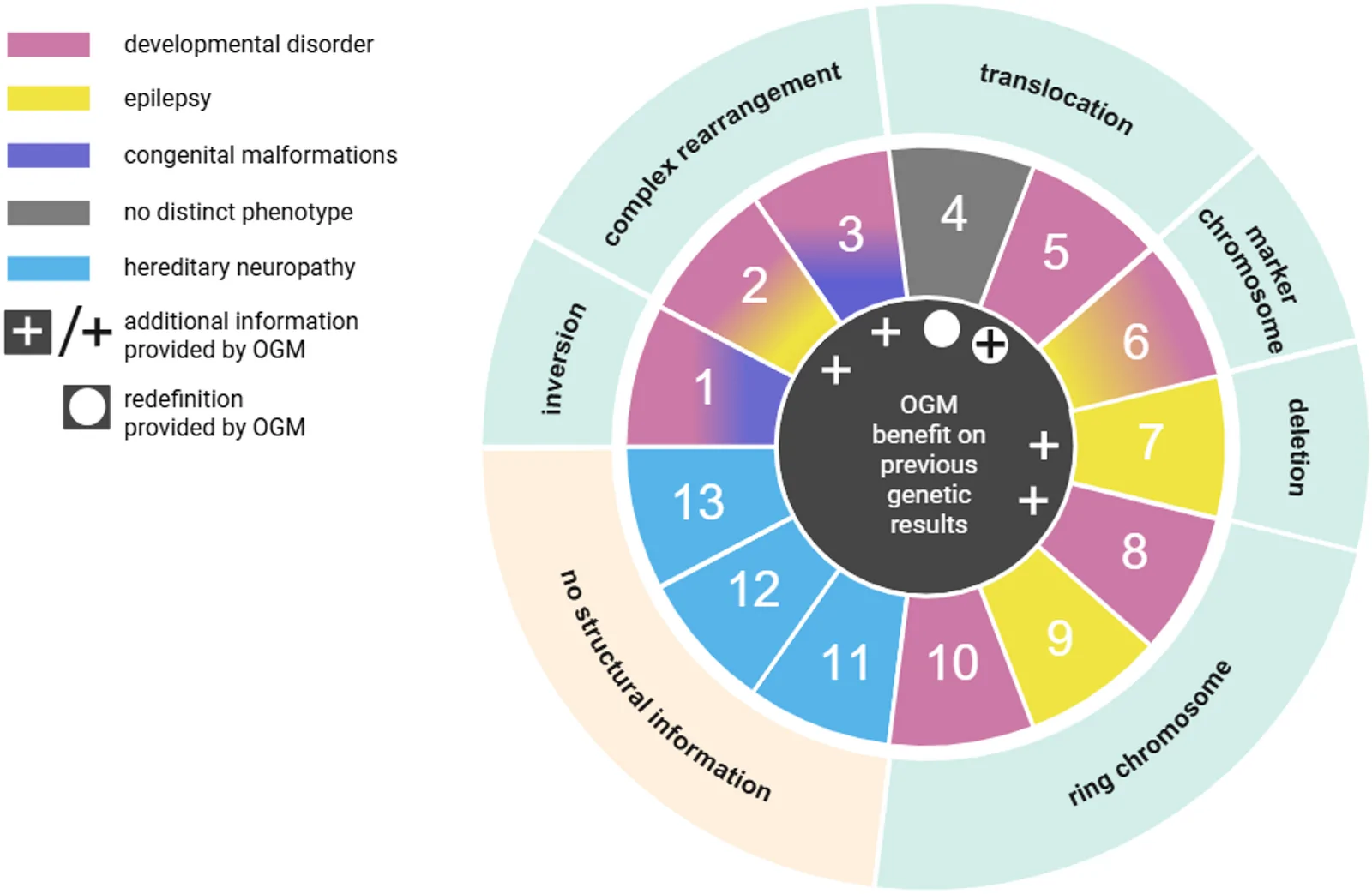
Overview of analyzed patient samples. Patient numbers (numbering 1–13) include color-coded clinical phenotype(s), previous structural classical cytogenetics results (outer ring) as well as overall evaluation of OGM results (middle circle including symbols). Dot and/or cross in the center circle indicate in which samples OGM either provided additional information or led to a redefinition of the previous diagnosis. A detailed overview of the corresponding results is provided in Supplementary Document 1, Table 4. Created in BioRender. Gerding, W. (2026). BioRender.com/2k1jxv3

Patients with neurogenetic or syndromic phenotypes and likely pathogenic aberrations exhibited different types of aberrations: translocations (two patients), inversion (one patient), deletion (one patient), complex rearrangements (two patients), ring chromosomes (three patients) and a supernumerary marker chromosome (one patient). The remaining three patients showed clinical signs of neuropathy, with inconclusive results from previous genetic testing. Overall patient information is summarized in Fig. 1. Detailed results of previous diagnostic workup including karyotyping, fluorescence in situ hybridization (FISH), CMA, NGS, multiplex ligation-dependent probe amplification (MLPA) or other genetic tests are provided in Supplementary document 1, Table 1. Using OGM in these patients (de novo pipeline, filtered at a frequency of ≤ 1% compared to the Bionanogenomics control population), on average 51.31 SVs were detected per sample. The most common type with more than 50% of all SVs detected are deletions, with a mean of 26.9 deletion SVs per sample. SVs that overlapped with known disease genes based on OMIM database data (https://omim.org) averaged 17.9 per sample. A detailed overview of SV results is available in Supplementary document 1, Table 1. To systematically analyze SV output for patient cases, results were categorized into three groups: ‘Concordance’ refers to SV results where OGM confirmed previous findings. ‘Redefinition’ refers to redefined karyotyping results after OGM analysis. Here, SVs confirmed previous results but gave additional genetic information (e.g. more detailed localization of the SV). The category ‘additional information’ includes novel SV results where OGM identified variants not detected previously, providing novel information. In the following, patients are introduced by genetic aberration type after standard genetic workup. Additional findings beyond the main aberration(s) were reported only when the affected genes or regions showed possible overlap with the patient’s phenotype or could potentially contribute to the phenotype.

### Inversion—patient 1

**Fig. 2:**
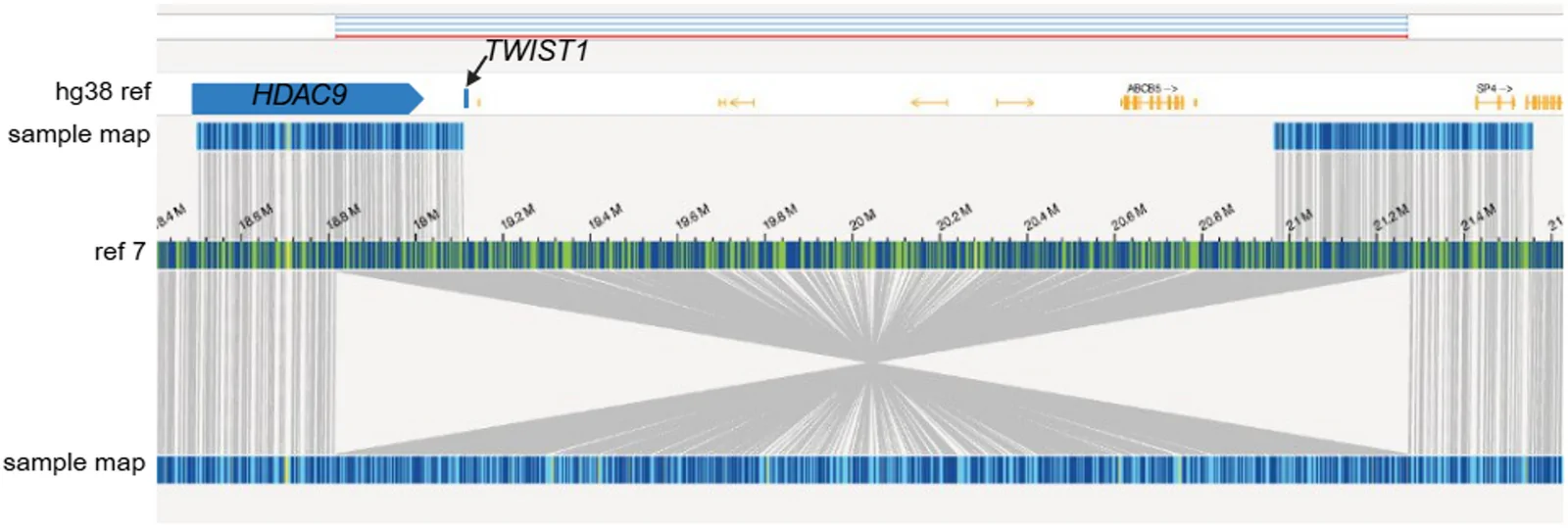
A large inversion disrupts *HDAC9* on 7p21.1 in patient 1. The figure shows the inversion identified through the de novo pipeline. Two smaller sample maps below the hg38 reference suggest that the inversion is present in heterozygous state. Created in BioRender. Gerding, W. (2026). Biorender.com/qy5v3em

Individual 1 is a 3-year-old girl with global developmental delay, hypotonia, a complex malformation of the cervical spine, a soft palate cleft and dysmorphic features (for clinical details, see Supplementary document 1, Table 5). Short-read WGS identified a heterozygous inversion disrupting the *HDAC9* gene, which has recently been associated with auriculo-condylar syndrome type 4 (Romanelli Tavares et al. 2022). This syndrome is characterized by malformed ears, round face, micrognathia, and malocclusion, among other features (Li et al., 2023). Additionally, WGS revealed a de novo variant of unknown significance in the *DDX3X* gene, presenting with a variant allele frequency (VAF) of ~ 0.30. OGM confirmed the inversion on chromosome 7p21.1-p15.3 (~ 18,818,327 to ~ 21,270,150 bp), spanning ~ 2.45 Mb in total (see Fig. 2). The inversion disrupts the *HDAC9* gene at breakpoint region 7p21.1 between exons 16 and 17, while there is no gene affected at the breakpoint region at 7p15.3. Using the RV pipeline, which provides precise information on the VAF, the inversion is displayed with a VAF of 0.31. The VAF of the inversion in WGS was not determined, however it is notable that the VAF value of the inversion detected by OGM closely aligns with the VAF of the *DDX3X* variant. This reveals similar VAF values for both genetic alterations, although these values do not report single cell frequencies. Unfortunately, we could not further evaluate these results since there was no additional material available for testing. OGM analysis of the patient’s parents confirmed a de novo aberration. In summary, OGM was able to confirm the inversion with high accuracy and, using the RV pipeline, determine the VAF.

### Inversion/complex rearrangement—patient 2

Individual 2 is a 12-year-old female presenting with severe intellectual disability and an autism spectrum disorder. Routine trio exome sequencing revealed an approximately 2.8 Mb de novo inversion on chromosome 2q24.3, with one breakpoint presumably located in intron 6 of the *KCNH7* gene, and the other in exon 7 of the *SCN2A* gene. A breakpoint PCR using primers located close to the aberrant reads detected by exome sequencing confirmed the inversion on 2q24.3 in a heterozygous state. The *SCN2A* gene is associated with several conditions, including developmental and epileptic encephalopathy 11 (OMIM #613721, autosomal dominant), episodic ataxia type 9 (OMIM #618924, autosomal dominant), and benign familial infantile seizures 3 (OMIM #607745, autosomal dominant), while there is no known disease-association for the *KCNH7* gene to date.

**Fig. 3:**
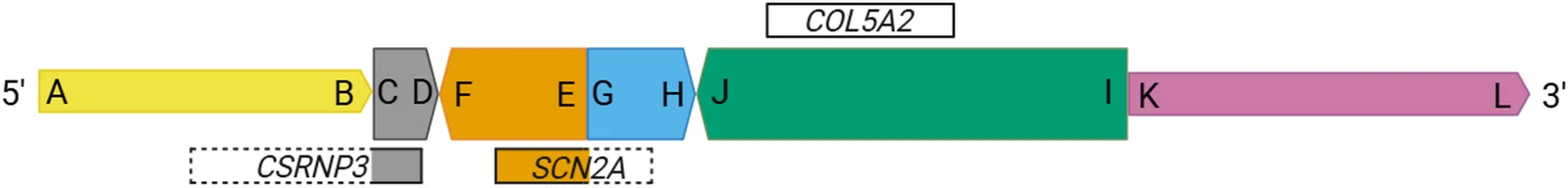
Schematic representation of the complex rearrangement map as visualized by the guided assembly pipeline. The map starts with the regular positioned segment AB (~ 188,534,809 to ~ 188,838,092), followed by the chromosomal rearrangement starting with the inserted segment CD (~ 165,453,379 to ~ 165,511,484) that partially contains the *CSRNP3* gene (regular position of this gene at 2q: hg38 chr 2:165,469,698–165,689,407, NM_001172173.2). The segment FE (~ 165,449,138 to ~ 165,313,330) is inverted and includes the *SCN2A* gene. Both genes, *CSRNP3* and *SCN2A* are disrupted by the rearrangement. The segments CD and FE are inserted from an upstream position on the same chromosome towards the telomeric direction at 2q. Segment GH (~ 189,250,139 to ~ 189,346,021) follows in regular orientation, while segment JI (~ 189,247,949 to ~ 188,843,234), which contains the *COL5A2* gene, is inverted. Segment KL (~ 189,350,314 to ~ 189,656,058) adjoints the rearrangement as a regular part of the map with proper orientation. *KCNH7*, with its original position in centromeric direction at 2q is not part of the shown map result generated by Bionanogenomics software. Gene positions are displayed above or below the rearranged segments, and their original sizes are represented by dashed outlines. Segment orientations are indicated by arrow direction. Created in BioRender. Gerding, W. (2026). BioRender.com/9vlbuo2

Surprisingly, OGM analysis with the de novo pipeline did not confirm a large inversion, but rather detected an accumulation of SVs in this region, with impacts on *SCN2A, CSRNP3* and *KCNH7*. Specifically, a ~ 27,016 bp insertion was identified between exons 6 and 7 of *KCNH7*, a deletion and an overlapping inversion affecting *SCN2A* and *CSRNP3,* as well as two intrachromosomal fusions affecting both these genes*.* Subsequent analysis using the guided assembly pipeline in Access 1.8.1 and Solve 3.8.1 revealed a complex rearrangement, as shown in Fig. 3. A segment of *SCN2A* was found to be inverted (~ 165,449,138 to ~ 165,313,330 bp, F-E) and together with a part of the adjacent *CSRNP3* gene (~ 165,453,379 to ~ 165,511,484 bp, G-H), translocated to an upstream position (~ 189,250,139) directly preceding an inverted segment containing the *COL5A2* gene (~ 189,247,949 to ~ 188,843,234 bp, J-I). Haploinsufficiency of *COL5A2* has been associated with classical Ehlers-Danlos syndrome, a subtype of Ehlers-Danlos syndrome (OMIM # 130,010). The insertion in the *KCNH7* gene, spanning from ~ 162,494,008 to ~ 162,501,238 bp appears to be separate from the complex rearrangement. An overview of this complex rearrangement is provided in Fig. 3.

### Complex rearrangement—patient 3

Individual 3 is a 2-year-old male with global developmental delay, syndromic facial features and bilateral vocal cord paralysis resulting in stridor, swallowing difficulties and poor feeding after birth. CMA analysis revealed three hemizygous copy number gains on chromosome X (arr[GRCh37**]**Xp21.3(24,967,800_25,522,422) × 2mat, Xp21.2(29,371,415_29,511,419) × 2mat, Xp21.2(29,763,147_29,913,688) × 2mat) also present in heterozygous state in the mother. They were formally classified as variants of unknown significance but suspected to be pathogenetically relevant. Additionally, a paternally inherited copy number loss on chromosome 4p13 as well as a de novo copy number gain on chromosome 6p22.2 of uncertain clinical significance were identified. Trio exome sequencing did not reveal a pathogenic variant.

We performed OGM in this male patient to characterize the three duplications on the X-chromosome using the de novo pipeline. OGM recognized four copy number gains on the p-arm of the X-chromosome: copy number gain A-B spanning from ~ 24,951,125 to ~ 25,156,665 bp (copy number: ~ 1.8), copy number gain C-D from ~ 25,236,147 to ~ 25,504,525 bp (copy number: ~ 2.1), copy number gain E–F from ~ 29,351,379 to ~ 29,491,328 bp (copy number: ~ 1.3) and copy number gain G-H from ~ 29,738,671 to ~ 29,894,441 bp (copy number: ~ 1.9).

**Fig. 4:**
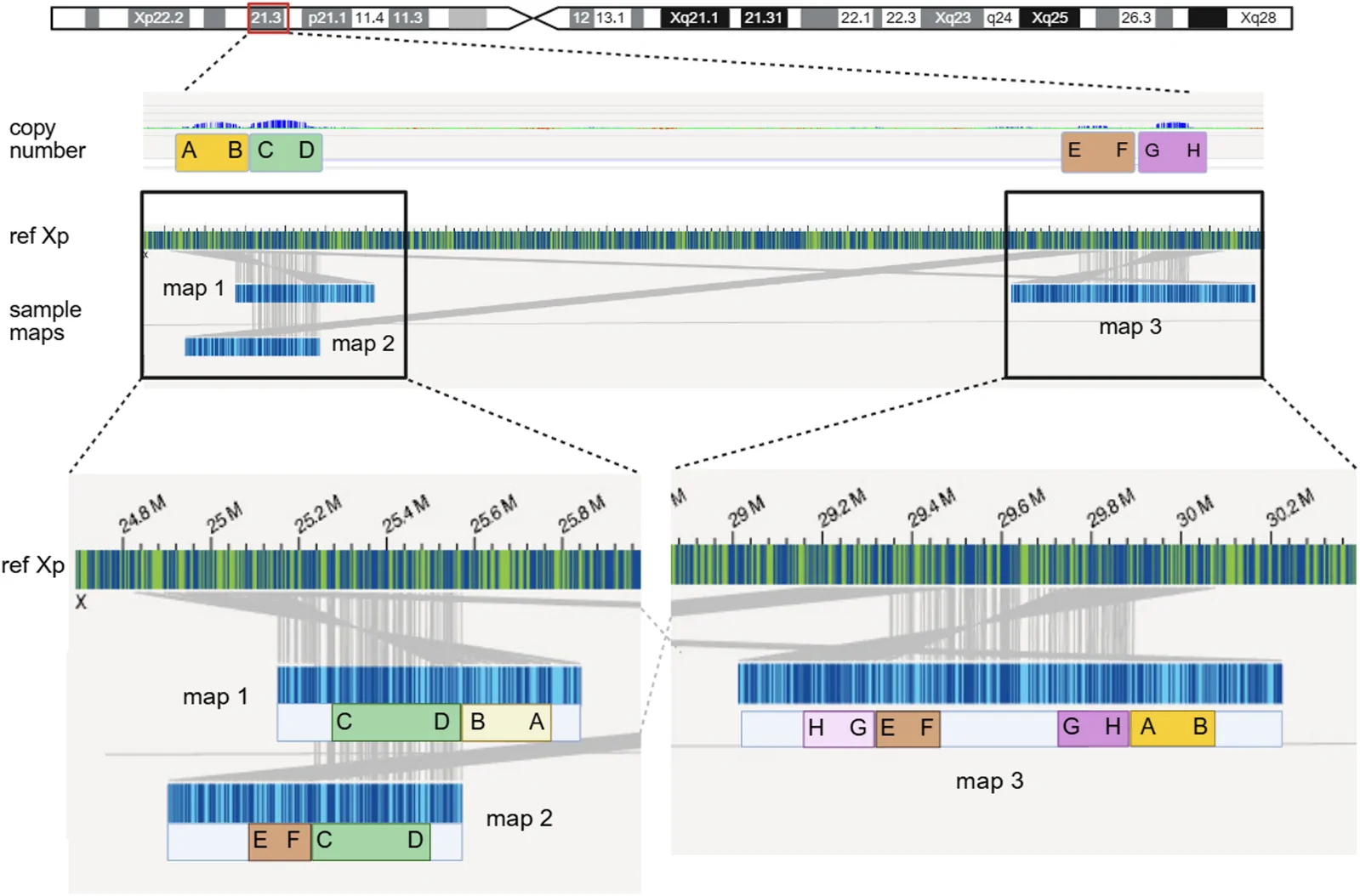
Complex rearrangement in patient 3 on chromosome X presumably due to a chromoanasynthesis event was visualized using the guided assembly pipeline. Four copy number gains are visualized by blue peaks. The SV pipeline shows three corresponding maps in this region. SV maps reveal complex rearrangements, as illustrated in two magnified views at bottom. Copy number gains and their localization and orientation are visualized as color boxes with orientation information using letters A–H. Created in BioRender. Gerding, W. (2026). BioRender.com/ldl31ji

These gains detected by OGM correspond to the regions identified by CMA. For detailed analysis of this region, additional analysis using the guided assembly pipeline (Bionanogenomics) was performed with Access 1.8.2 and Solve 3.8.2, respectively. This enabled a detailed structural characterization of this region and revealed a complex rearrangement, visualized as three maps. Because of the complex nature of this structural formation including DNA copy-number changes and particularly region-focused duplications and triplications, this rearrangement is presumably the result of a chromoanasynthesis event (Lee et al. 2007; Liu et al. 2011). The structural localization of duplicated regions and their respective orientation is depicted in Fig. 4.

This complex rearrangement affects the *POLA1*, *ARX* and *ILRAPL1* genes. The *POLA1* gene is associated with X-linked pigmentary disorder (OMIM #301220) and X-linked Van Esch-O'Driscoll syndrome (OMIM #301030). The *ARX* gene is linked to multiple X-linked conditions, including developmental and epileptic encephalopathy 1 (OMIM #308350), hydranencephaly with abnormal genitalia (OMIM #300215), intellectual developmental disorder 29 (OMIM #300419), lissencephaly 2 (OMIM #300215), Partington syndrome (OMIM #309510) and Proud syndrome (OMIM #300004). Additionally, the *IL1RAPL1* gene is associated with X-linked intellectual developmental disorder, 21 (OMIM #300143). These affected genes could potentially contribute to the patient's phenotype. However, X inactivation analysis in the mother showed no evidence of skewed inactivation, and other male family members from the maternal side were not available for analysis in order to confirm or exclude the pathogenetic relevance of this rearrangement in male patients. Additionally, OGM detected a deletion in the *CTCF* gene on chromosome 16, affecting exons 1 and 2. *CTCF* is associated with autosomal dominant intellectual developmental disorder 21 (OMIM #615,502) with speech and motor delays, including dysphagia and hypotonia, which could align with the phenotype of the patient, but vocal cord paralysis is not typically associated (Valverde de Morales et al. 2024). The deletion on chromosome 4 identified by CMA was detected by OGM (4p13, ~ 145 kb), whereas the duplication on chromosome 6 (6p22.2, ~ 160 kb) was most likely missed by the Bionnanogenomics SV algorithm. In summary, OGM was able to characterize a complex rearrangement in this sample of patient 3 that could not be resolved by previous genetic tests, thereby providing additional information.

### Translocation—patient 4

**Fig. 5:**
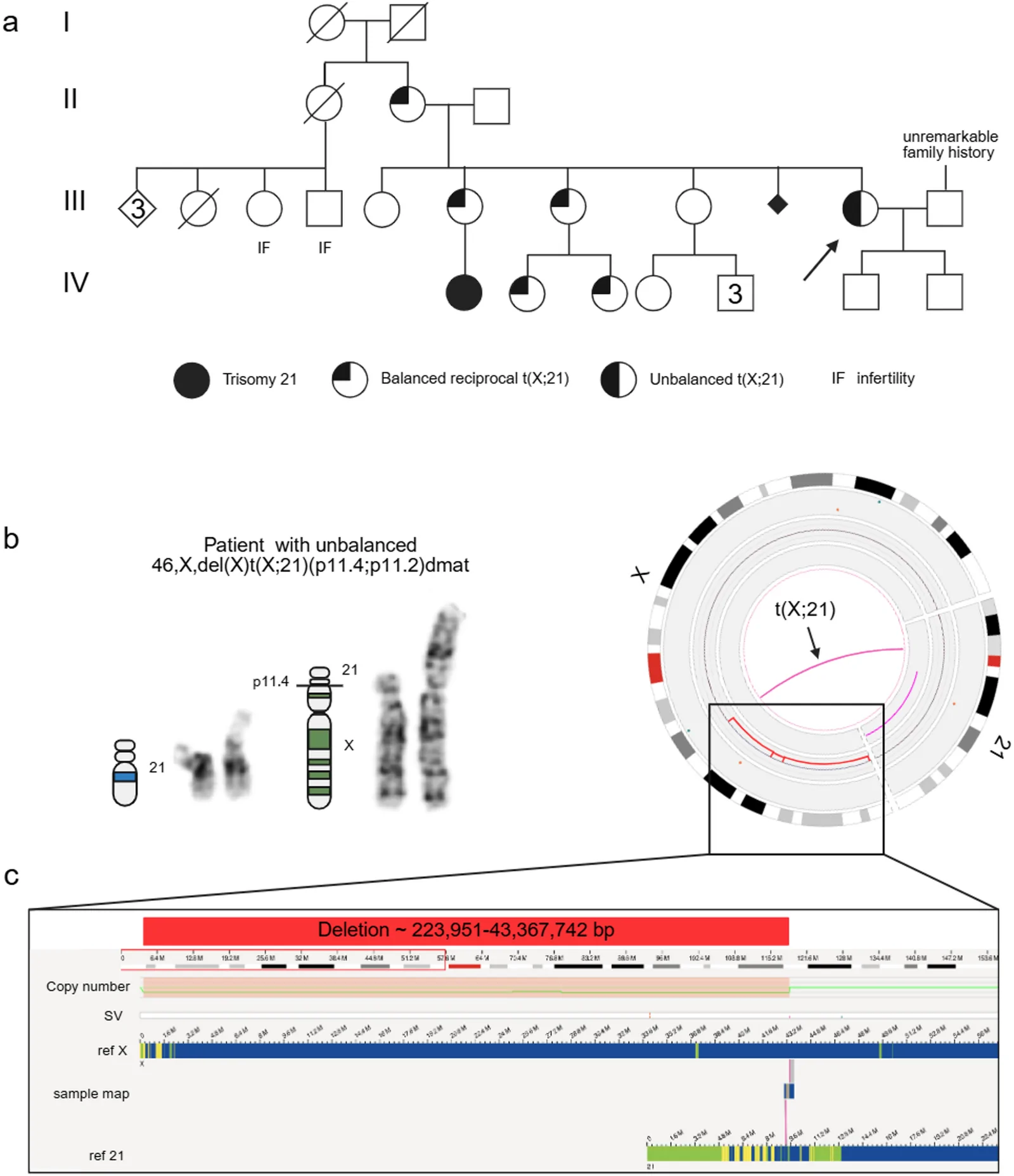
Characterization and refinement of unbalanced translocation t(X;21) in patient 4.** a** Pedigree of the family, the index patient 4 is indicated by an arrow. ** b** Ideograms and chromosomes after karyotyping are depicted for t(X;21). The deletion on Xp results in a significant shortening of the p-arm visualized by karyotyping, also visible as a large, deleted region (detected by CNV pipeline) depicted in detail in (** c**). Created in BioRender. Gerding, W. (2026). Biorender.com/u6a0sib

Individual 4 is a 44-year-old female with a positive family history of a balanced translocation t(X;21) in her mother and two sisters; one sister has a daughter with trisomy 21 (Fig. 5a). She requested testing for the familial translocation because of potential future reproductive plans of her two sons. Except for a below-average height of 1.50 m and a kidney reflux surgically treated in childhood, she does not display any symptoms. However, karyotyping revealed an unbalanced translocation (46,X,del(X)t(X;21)(p11.4;p11.2)dmat) with a monosomy of a large part of Xp and a partial trisomy of 21p (short part of satellite stalks and satellite itself). Analysis of X-inactivation showed a skewed inactivation pattern of 95%: 5% in favor of the unaffected X chromosome, potentially explaining the rather mild phenotype. Based on the karyotyping result, a more thorough characterization of the deleted regions was recommended.

OGM confirmed the deletion on Xp starting from Xp11.3, redefining the previous breakpoint reported at Xp11.4 by karyotyping (Fig. 5b, c). The translocation was initially not detected by OGM using standard analysis settings. This is most likely because the breakpoint on chromosome 21 is located on the short arm, which is technically excluded from OGM standard analysis. The short arms of acrocentric chromosomes contain a high density of segmental duplications (segdupes), gaps, and chromosomal regions shared with other acrocentric chromosomes (Guarracino et al. 2023). Application of modified filter settings displaying all SVs, removal of masked regions and enabling view of all translocations (instead of standard settings) were applied in the software and t(X;21) was identified with low coverage of 3x. According to Bionano Solve Theory of Operation this translocation was identified as “common_translocation”, describing false-positive translocations in regions difficult to analyze (Bionano Genomics 2021b) observed in 10 or more samples in the control database. The deletion on the X chromosome is displayed as a copy number loss in three segments and an absence of heterozygosity/loss of heterozygosity (AOH/LOH) region, a genomic region where both alleles are identical or one allele is missing: ~ 223,951 to ~ 24,858,179 bp, 24,862,420 to ~ 28,121,474 bp, and 28,127,751 to ~ 43,367,742 bp. Combined, this represents a loss of approximately ~ 43 Mb on one of the X chromosomes, as the deletion likely extends to the terminal end from 0 to ~ 43,143,791 bp, with the gaps in between corresponding to masked regions in the analysis software. This result aligns with and further redefines the previous findings from karyotyping.

### Translocation—patient 5

Individual 5 is a 10-year-old boy with developmental delay, borderline intellectual functioning (IQ80), attention deficit disorder, tall stature and obesity. Karyotyping revealed a de novo balanced translocation 46,XY,t(5;6)(q13;q23), which was confirmed by FISH, but no copy number change in CMA analysis. OGM analysis confirmed the translocation between chromosomes 5 and 6 and redefined the breakpoint region to 46,XY,t(5;6)(q14;q24). While the identified breakpoint region on chromosome 6 did not encompass any known genes, this chromosomal rearrangement led to the disruption of the *RASGRF2* gene located on chromosome 5, which plays an important role in the MAPK pathway. Disruptions in different components of this pathway are causative for various developmental disorders, making it a candidate gene for this patient's developmental delay (Sweatt and Weeber 2003). In this case OGM led to the identification of a new potential candidate gene for developmental disorders. Detailed information on this patient case has already been reported (Lederbogen et al. 2024).

### Supernumerary marker chromosome—patient 6

Patient 6 is an 11-month-old girl with global developmental delay, muscular hypotonia and epilepsy in form of infantile spasms. Karyotyping identified a supernumerary marker chromosome derived from chromosome 15, consistent with idic(15) syndrome, leading to tetrasomy of 15q. CMA analysis revealed a quadruplication of the 15q11.2-13.1 region along with a triplication of the 15q13.1-13.2, and additionally showed a 175 kb deletion on chromosome 7q32.1, which was also present in the unaffected mother.

**Fig. 6:**
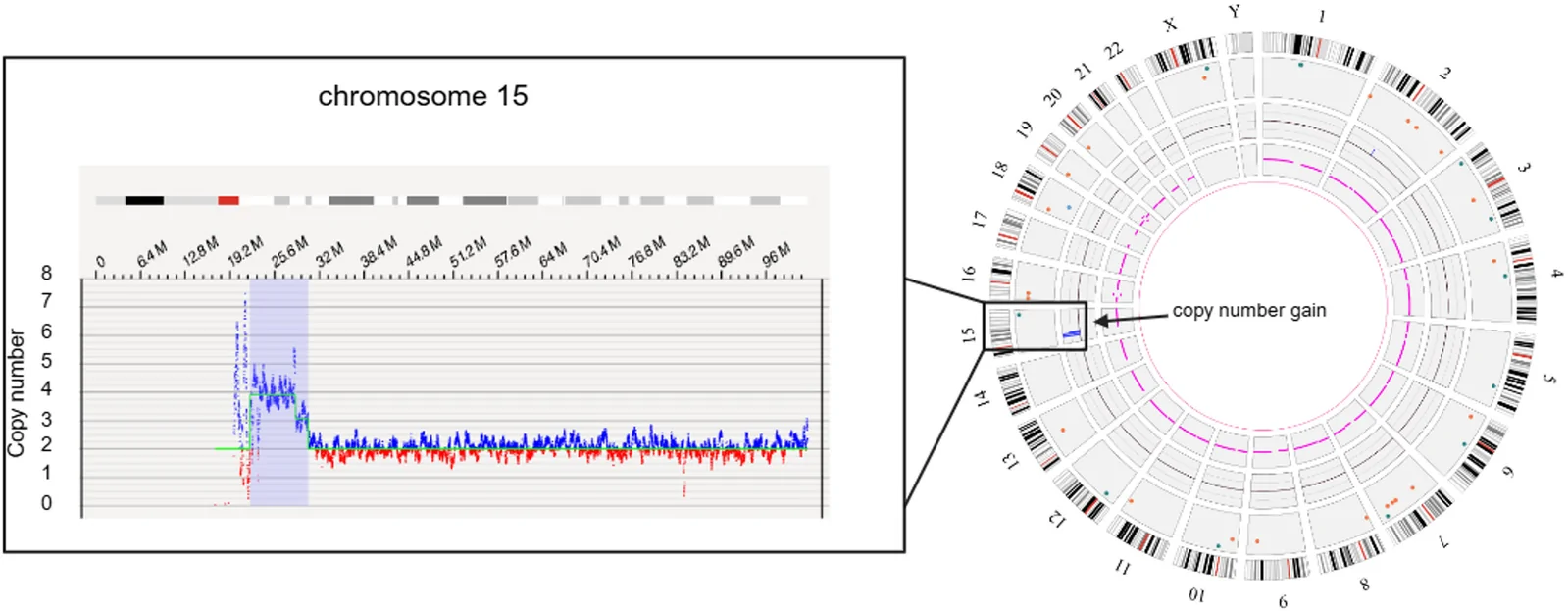
OGM analysis in a patient with marker chromosome 15 using the de novo pipeline. The left side displays the copy number profile for chromosome 15 in the Access software: an increase in copy number is observed in the highlighted region, representing the marker chromosome. On the right side, the analysis result is visualized in the circos plot view. Created in BioRender. Gerding, W. (2026).Biorender.com/wabwl6j

OGM confirmed CMA results for chromosome 15q11.2-q13.2, in two regions: ~ 22,049,837 to ~ 28,584,463 bp (~ 6.5 Mb) with a copy number of 4, and ~ 28,589,702 to ~ 30,418,315 bp (~ 1.8 Mb) with a copy number of 3. This is consistent with an asymmetric idic(15) (Fig. 6). Typically, isodicentric chromosomes appear in OGM with one duplicated and one deleted chromosome arm. Inverted duplications in marker chromosomes mirror the presence of two identical arms created by a fold back configuration (Zhang et al. 2023). However, in this case, the idic(15) could not be visualized in OGM, primarily because the double centromere cannot be resolved due to OGMs limited resolution in centromeric regions (Barseghyan et al. 2024). Because chromosome 15 is acrocentric, there were very few labels on the p-arm. As a result, a potential deletion in this region was not detected by OGM. Additionally, three further aberrations were detected by OGM: a deletion on chromosome 2, affecting exons 5–8 of the *MYT1L* gene, which is associated with intellectual developmental disorder, autosomal dominant 39 (OMIM #616,521), and the deletion also found by array analysis on chromosome 7 (128,701,890–128,878,768 bp), affecting multiple genes, including *OPN1SW* and *FLNC. OPN1SW* is associated with colorblindness, tritan (OMIM #190,900, autosomal dominant), and *FLNC* is linked to several myopathies and cardiomyopathies, including myopathy, distal, 4 (OMIM #614,065, #617,047, #609,524, autosomal dominant). Lastly, a deletion was found on chromosome 18, affecting exon 4 of the *PIEZO2* gene, associated with arthrogryposis, distal, type 3 and 5 (OMIM #114,300, #108,145, autosomal dominant), although this condition is typically linked to gain-of-function mutations (Alper et al., 2017). In summary, OGM was able to detect and visualize the quadruplication and triplication in this case. However, due to the acrocentric nature of the chromosome and its resolution limitations in the centromeric region, the isodicentric chromosome could not be visualized.

### Deletion—patient 7

Individual 7 is a 3-year-old male with four complicated febrile seizures and delayed speech development. Karyotyping and Fragile X-testing in our patient gave normal results. Panel testing for hereditary epilepsy revealed a 7 kb microdeletion in the 2q24.3 region, likely affecting exons 21–24 of the *SCN9A* gene; however, further confirmation with a different method was recommended. Array analysis confirmed the heterozygous deletion in 2q24.3, affecting intron 20 of the *SCN9A* gene, though with very limited resolution as it was detected by only one probe.

OGM analysis confirmed the deletion from ~ 166,211,549 to ~ 166,238,439 bp and a size of ~ 26.9 kb, suggesting that exons 20–24 are involved. *SCN9A* is associated with different disorders affecting pain perception and nerve function (OMIM #243,000, #167,400, #133,020), but findings regarding the association of *SCN9A* with epilepsy are contradictory (Mulley et al. 2013; Brunklaus 2021). Subsequently, OGM analysis of the patient`s parents revealed that he inherited the *SCN9A* deletion from his healthy father, suggesting that it may rather not be disease-causing in our patient. In summary, while the cause of the febrile epilepsy in the patient remains unknown, the *SCN9A* deletion was clearly identified in both the father and son using OGM and therefore aiding in the diagnostic process.

### Ring chromosome—patient 8

Patient 8 is a 4-year-old girl with global developmental delay (non-verbal IQ: 65) and dysmorphic features including astigmatism. CMA analysis revealed three aberrations on chromosome 18: a terminal deletion at the end of the p-arm (920 kb, 17 genes), an adjacent duplication (243 kb), and a terminal deletion at the end of the q-arm (12.2 Mb, 80 genes), hinting to the presence of a ring chromosome 18 (r(18)). Karyotyping confirmed the presence of a r(18) in 50 examined metaphases and a double ring configuration with two r(18) in two cells.

**Fig. 7:**
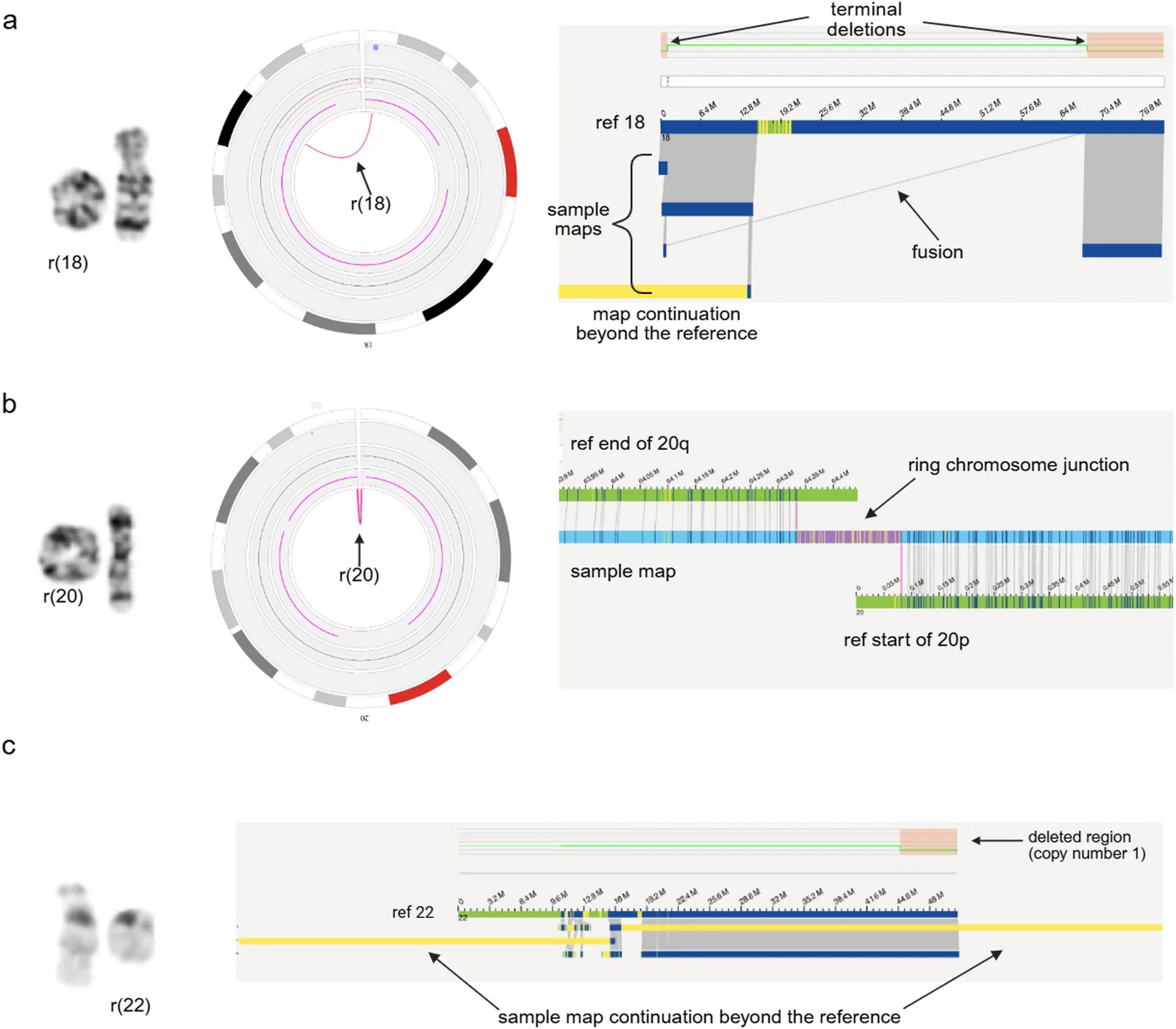
Ring chromosome structural analysis in karyotyping and OGM using the de novo pipeline. On the left side, ring chromosomes from karyotype analysis are displayed for patients 8, 9 and 10. a) Patient 8: The circos plot in the center shows the r(18). On the right, the copy number pipeline demonstrates terminal deletions on both arms of chromosome 18. Two maps indicate a fusion between the ends of p- and q-arms, along with map continuity beyond the reference maps of 18p. b) Patient 9: The circos plot displays a r(20), indicated by the arc in the inner circle. On the right, the end of chromosome 20q and the beginning of 20p are fused, confirming ring formation. c) Patient 10: The maps extend beyond the reference region, indicating the presence of a r(22). The p-arm (in green) does not show any labels, the q-arm displays a deletion from 22q13.31 to 22q13.33 (~ 5.83 Mb). Created in BioRender. Gerding, W. (2026). BioRender.com/7wvdp82

OGM confirmed the presence of a r(18), after adjustment of SV confidence for “Intra-Fusions” from “recommended” to “all”, unmasking all intrachromosomal fusions (Fig. 7a). In addition, the sample was also analyzed using the RV pipeline, where two ring chromosome fusion sites were identified directly with standard filter settings: one between p11.32 and q22.1 (VAF 0.34) and another between p11.31 and q22.3 with low data depth (VAF 0.1; coverage 2X), potentially indicating the double-ring formation found in two out of 50 cells. OGM further confirmed the presence of terminal deletions on chromosome 18, including a ~ 12.18 Mb deletion on the q arm (~ 68,071,855 to ~ 80,256,374) and a ~ 1.02 Mb deletion on the p arm (~ 18,868 to ~ 1,041,105), likely starting from pter. The latter was detected after removing the CNV masking filter. Additionally, three inverted duplications were identified at the ring fusion site at 18p11.32. These duplications varied in size, with the longest one representing the complete duplicated region (~ 1,065,799 to ~ 1,266,759 bp; ~ 200.96 kb), as the maps for the shorter ones were too small to display full extent of the duplication. The combination of deletion and inverted duplication is common in ring chromosomes (Rossi et al. 2008), but the inversion had not been detected in previous genetic tests. Lastly, a deletion in *CUX2* (Chr. 12, exon 19), linked to developmental and epileptic encephalopathy 67 (OMIM #618,141, autosomal dominant) was found that could be related to the patient`s phenotype although the patient has not shown seizures yet.

### Ring chromosome—patient 9

Individual 9 is a 29-year-old male with temporal lobe epilepsy starting from the age of 14, followed by a decline in cognitive function and psychotic symptoms. Karyotyping identified a r(20) in mosaic state in 14 out of 20 metaphases, which has been described as causative for a very rare form of epilepsy (Daber et al. 2012). CMA as well as a panel analysis for epilepsy did not reveal other pathogenetically relevant variations, except for a maternally inherited variant of uncertain significance (c.1970 T > C, p(Val657Ala)) in the *SCN3A* gene.

OGM detected the r(20) only after visualization of all structural variants, including those masked by the software (see Fig. 7b). This required unmasking results by adjusting “general SV filters” to “all structural variants”. The results did not indicate a terminal deletion, especially not in the epilepsy-associated genes *CHRNA4* and *KCNQ2*. Additional findings in OGM included a duplication of unknown zygosity on chromosome 22q11.22 spanning about ~ 226.972 kb (~ 21,956,748- to ~ 22,222,820 bp), which includes the *TOP3B* gene. This gene has been described in the literature as potentially playing a role in the pathogenesis of different diseases, including epilepsy (Daghsni et al. 2018). However, a potential contribution of this aberration to the patient´s phenotype remains speculative.

### Ring chromosome—patient 10

Individual 10 is a 37-year-old woman with a global developmental disorder, intellectual disability, absent speech, syndromic facial features, impaired vision, chronic lymphedema and pleural effusions as well as recurrent erysipelas. Karyotyping identified a r(22) in all metaphases, with a deletion in the 22q13.33 region, which was confirmed by FISH. CMA confirmed the deletion on chromosome 22, extending it to 22q13.31q13.33 encompassing ~ 5.83 Mb and approximately 81 genes. The deletion includes the *SHANK3* gene, leading to the diagnosis of Phelan-McDermid syndrome (PMS), which is caused either by a deletion in the 22q13.2–33 region or by a pathogenic variant in the *SHANK3* gene (Schön et al. 2023). Additionally, CMA identified a gain of 18 genes on chromosome 5q32 with unknown significance, including the *CAMK2A* gene associated with autosomal dominant intellectual developmental disorder type 53 (OMIM, #617798).

In this patient, OGM detected a heterozygous deletion on chromosome 22q13.31-13.33 (~ 44,979,409 to ~ 50,805,587 bp; ~ 5.83 Mb). While no ring chromosome was formally called, the presence of a ring structure was suggested by OGM maps extending far beyond the reference (see Fig. 7c). The p-arm of chromosome 22 displayed very few labels. Subsequent OGM analysis using the T2T reference genome, which includes more labels than the hg38 reference, also did not result in a detection of a ring formation. The sparse labeling on the p-arm on chromosome 22 most likely impaired the direct identification of the ring chromosome. A previously identified copy number gain at 5q32 in CMA analysis was called as a duplication split event by OGM, disrupting the *CAMK2A* gene between exons 2 and 3. This disruption suggests a potential contribution to the patient's developmental disorder. Lastly, a small deletion on chromosome 5 affecting exon 5 of the *CDH18* gene was identified with OGM that had not been discovered in CMA analysis. This gene has not been described as causative for a monogenic disorder but associated with psychological diseases in genome-wide association studies (Hawi et al. 2018). Whether this SV could contribute to our patient`s phenotype remains unclear.

### Workflow for structural evaluation of ring chromosomes by OGM

**Fig. 8:**
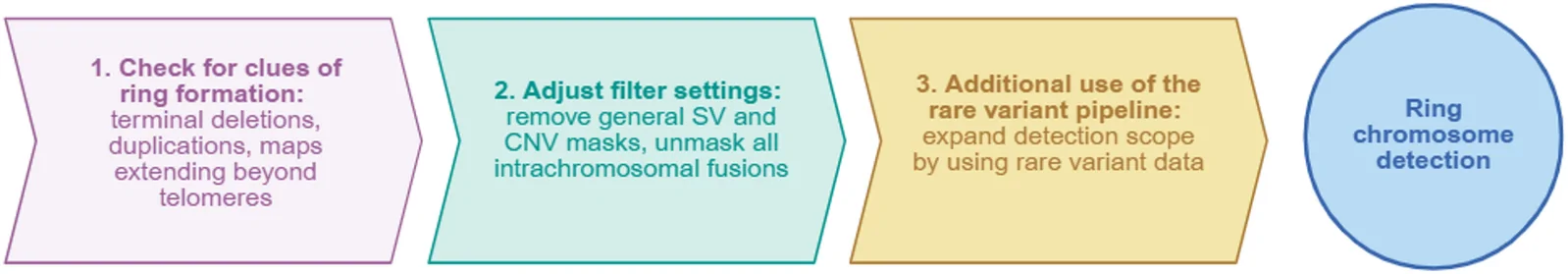
Schematic representation of suggested analysis workflow for ring chromosome analysis using OGM. Due to reduced sensitivity at terminal chromosome regions, it can be beneficial to enable ring formation detection by adjusting standard filter settings, remove masks and use the RV pipeline. Created in BioRender. Gerding, W. (2026). BioRender.com/4i07zow

Based on the evaluation of the three abovementioned patients with ring chromosomes, a workflow for the evaluation of ring chromosomes using OGM was developed. Based on our cases, typical indicators for a ring chromosome are terminal deletions, adjacent duplications, fusion of maps between the p and q-arm and maps extending beyond the reference regions. Following the inspection of these indicators, we suggest adjusting standard filter settings. In our cases, (1) changing masking filter under “general SV filters” from “non masked structural variants only” to “all structural variants” and (2) unmasking of the copy number variant masking filter in order to remove potentially masked terminal deletions was useful. In addition, we suggest: (3) SV confidence for “Intra-Fusions” should be changed from “recommended” to “all” (defined under “Filter by SV type” settings). One ring chromosome was only seen in combination of all these filter changes; therefore, different combinations of adjustment should be applied. Finally, an additional analysis using the RV pipeline might reveal the presence of a ring chromosome, as in our study. A schematic overview for a suggested workflow is provided in Fig. 8.

### Neuropathies without causative variants in preceding routine analyses (Patients 11, 12 and 13)

This additional group of individuals comprises patients with suspected hereditary neuropathy for whom no genetic cause could be identified with routine diagnostics. Patient 11 is a 10-year-old girl with motor delay, stiff gait, clubfoot deformities and reduced nerve conduction velocity (NCV); OGM analysis did not reveal any pathogenetically relevant SV or CNV. In patient 12, a 56-year-old male with slowly progressive gait problems, hypesthesia, elevated creatine kinase (CK) levels and a positive family history with an equally affected mother and son, OGM revealed a duplication involving exons 21–25 of the *PIK3C3* gene. Dysregulation of PI3P has been hypothesized to cause Charcot-Marie-Tooth (CMT) neuropathy in mouse models (Logan et al. 2017). However, reevaluation of the NGS analysis showed that the duplication was also present in the patient’s affected son, but not in the affected mother. This suggests that the duplication is unlikely to be the causative familial aberration.

Individual 13 is a 31-year-old female with clinical symptoms of hereditary neuropathy and/or a connective tissue disorder, including a distal-dominant tetraparesis with atrophy and a continuous decline in motor function. OGM detected an insertion in the *EP400* gene, affecting exons 49–51 (Chr. 12). There is an indication that *EP400* deficiency in Schwann cells can lead to persistent expression of early developmental regulators and peripheral neuropathy (Fröb et al. 2019). However, the definitive disease linkage should be reevaluated in the coming years.

Taken together, none of the patients in the group of hereditary neuropathies showed a conclusive causative SV or CNV through OGM analysis.

## Discussion

OGM can offer additional insights into standard routine diagnostics like karyotyping or FISH. While karyotyping relies on analysis of banding pattern with a resolution of 5–10 Mb, OGM provides a resolution up to 20,000 times greater (Moore et al. 2023; Nilius-Eliliwi et al. 2023). Proof-of-principle studies demonstrated that high-resolution OGM could detect nearly all types of chromosomal aberrations with non-centromeric breakpoints, compared to standard methods (Mantere et al. 2021; Nilius-Eliliwi et al. 2023; Sahajpal et al. 2023; Barseghyan et al. 2024). In the present study, we evaluated the potential of OGM to better characterize and specify neurogenetic and syndromic cases that required further investigation after standard genetic testing. In 10 patients with different neurogenetic diagnoses, OGM confirmed all structural findings previously detected by karyotyping, array, and/or NGS. Furthermore, in five cases OGM provided additional insight into the structural aberrations that was not evident from the routine analysis. For example, in a patient with a balanced translocation, a novel candidate gene, potentially relevant for neurodevelopmental disorders was identified (for detailed information see (Lederbogen et al. 2024)). OGM also led to a redefinition of breakpoint regions in both a balanced (patient 5) and an unbalanced familial translocation (patient 4). Furthermore, three adjacent X-chromosomal duplications in a patient with a syndromic disorder could be characterized as a complex rearrangement on the X chromosome (patient 3), and for a r(18), OGM revealed an inverted duplication at the ring fusion site region (patient 8). For patient 2, the initial diagnosis of an inversion disrupting *SCN2A* and *KCNH7* was revised to a complex rearrangement involving additional genes. However, for some of the predescribed aberrations, including the three ring chromosomes, a translocation involving an acrocentric chromosome and a marker chromosome, modified filter settings were required in order to detect the aberrations, in case centromeric regions or acrocentric chromosomes were involved which are known to be prone for difficulties in OGM analysis (Mantere et al. 2021).

A particular focus of interest in the present study is the characterization of three ring chromosomes (involving chromosomes 18, 20 and 22). Ring chromosomes occur in approximately one in 50,000 newborns and show a high genetic heterogeneity including complete rings, terminal deletions, complex rearrangements and dynamic mosaicism, along with high variability of the clinical phenotype. Karyotyping is capable of identifying ring chromosomes, and FISH and CMA can be used to characterize regions of loss or gain associated with the ring structure. While NGS can determine the exact breakpoints, it can also fail to detect the presence of a ring chromosome (Li et al. 2022; James et al. 2024). OGM typically identifies ring chromosomes as a loss of subtelomeric regions with a p-arm to q-arm fusion, though it is important to note that unbalanced translocations with the homologous chromosome cannot be excluded (Moore et al. 2023). Published OGM data on ring chromosomes are so far rare and mostly embedded as single cases in a larger patient group. A recent study for example characterized a ring chromosome 17 using OGM, which was not fully resolved by conventional methods alone (Kim et al. 2025). In cancer studies, ring chromosomes have been identified in hematologic malignancies such as ALL, myelodysplastic syndromes, as well as in soft tissue and bone tumors (Yang et al. 2022; Sahajpal et al. 2022; Vieler et al. 2023; Baelen et al. 2024). On the other hand, in both preimplantation and prenatal genetic testing studies, OGM missed some cases of ring chromosome mosaicism (Levy et al. 2023; 张志强 et al. 2024). In one study, a ring chromosome 21 was characterized using the T2T reference genome, and in another large patient group, a mosaic ring chromosome X was reported in a patient with developmental delay and growth retardation (Mantere et al. 2021; Schuy et al. 2024).

In the present study, we demonstrate OGM findings for three individuals with ring chromosomes 18, 20 and 22, associated with the clinical diagnoses of global developmental delay, focal epilepsy and Phelan-McDermid syndrome, respectively. We found that all three previously known ring chromosomes were successfully recognized with OGM, but only after adjusting the routinely used filter settings. For example, the r(18) was initially not displayed using Bionano’s standard filters (< 1% in the reference population); however, when removing the intrachromosomal fusion and the CNV mask, the ring chromosome and the p-arm deletion became visible. On the other hand, using the RV pipeline, the ring chromosome was directly displayed by the software. Similarly, the r(20) was initially masked by the software as the fusion occurred in the telomeric region with minimal or no loss of chromosomal material, which is commonly masked due to gaps. Only after removal of SV masking filters it became visible. The r(22) was not shown as an intrachromosomal fusion as the other two. The difficulty in detecting the r(22) is most likely explained by the challenge OGM faces in accurately displaying the p-arms of acrocentric chromosomes (Barseghyan et al. 2024). Specifically, there were only a few labels in p11.2 and none in p12 and p13. Since karyotyping indicated that the ring chromosome fusion occurred at p13, where no labels were present, this complicated the detection. With the knowledge of the ring chromosome gained from prior cytogenetic testing, it became apparent, upon examining the whole chromosome in the genome browser, that the maps extended beyond the reference maps, delivering an indication of the ring structure.

Given our experiences from these three ring chromosome samples, we developed a strategy for the detection of ring chromosomes with OGM that is outlined in detail in the results section. This strategy involves searching for typical indicators for ring chromosomes, such as terminal deletions, adjacent duplications, fusion of maps between the p and q-arm and maps extending beyond the reference regions. The next step is adjusting the standard filter settings, so that routinely masked regions are displayed and can be individually evaluated. Finally, an additional analysis using the RV pipeline might reveal the presence of a ring chromosome. With these methods, the detection of ring chromosomes, even involving acrocentric chromosomes, by OGM appears feasible. However, replication in a larger cohort of patients with known ring chromosomes is needed in order to confirm our findings, and it cannot be excluded that ring chromosomes may be missed if OGM is applied as a first line test.

Ring chromosomes have also been described in Turner syndrome, and Mantere et al. described confirmation of a mosaic r(X) with OGM in a patient with developmental delay (Mantere et al. 2021; Fiot et al. 2022). In the present study, we did not have a patient with a r(X), but a female carrying an unbalanced X;21 translocation, which posed similar technical challenges as seen for the ring chromosomes involving acrocentric chromosomes. The translocation was initially not displayed as it occurred in a masked region of the p-arm of chromosome 21. With the prior knowledge of karyotyping, the standard filter settings could be adjusted to detect the translocation. The patient presents with a partial monosomy of the Xp arm and a partial trisomy of the 21p arm due to the inheritance of an unbalanced translocation from the mother, who carries a balanced X;21 translocation. The triplication of the 21p arm is clinically insignificant, as it mainly consists of inactive heterochromatin (Wyandt et al. 2017). The patient’s below average height may be explained by haploinsufficiency of the *SHOX*-gene, located on the deleted Xp arm (Oliveira and Alves 2011). X-inactivation analysis revealed preferential inactivation of the translocated X chromosome, which likely accounts for the minimal phenotypic impact despite the presence of multiple haploinsufficient genes in the deleted region. Similar technical limitations were observed in patient 6 with a supernumerary marker chromosome derived from acrocentric chromosome 15. The supernumerary marker chromosome was detected in OGM as a copy number gain but was not identified as a separate chromosome. Since chromosome 15 is acrocentric, the deleted arm could not be visualized, though the quadruplication and triplication of segments on 15q were correctly identified.

Our case series also included two patients in whom short-read WGS identified inversions. In patient 1, presenting with craniofacial malformations and developmental delay, OGM reliably confirmed an inversion disrupting the *HDAC9* gene. The breakpoint region detected by OGM correlates with the WGS result and presumably disrupts regulatory regions of the neighboring *TWIST1* gene, causative for craniosynostosis as well as auriculocondylar syndrome ((Romanelli Tavares et al. 2022). In contrast, the described inversion on chromosome 2q24.3 in patient 2, presenting with global developmental delay and epilepsy, was not confirmed as such. Instead, OGM revealed that the region was involved in a complex rearrangement including several intrachromosomal translocations and smaller inversions. The patient’s phenotype is most likely explained by the disruption of *SCN2A* due to the inversion and translocation. The adjacent gene *CSRNP3* is also affected, but has not been linked to intellectual disability or epilepsy (Akaboshi et al. 2024). The rearranged segment was further inserted near an inverted *COL5A2* gene, associated with classical Ehlers-Danlos syndrome (EDS), but the patient did not show major clinical features of EDS such as skin hyperextensibility or generalized joint hypermobility. To resolve the discrepancies between NGS results and OGM findings, long-read sequencing has been initiated. The outcome of this analysis will be reported separately and is not included in this study.

Similarly, in patient 3, showing a developmental disorder and congenital malformation, OGM revealed a complex rearrangement on the X chromosome underlying three maternally inherited duplications that had been detected by array analysis. By providing a detailed resolution of the genomic structure in this region, OGM enabled a detailed characterization of the region leading to the duplications. As demonstrated in a recent study, OGM can improve the interpretation of copy number gains by uncovering the underlying structural variants and has been shown to contribute to the reclassification of uncertain variants (Dharmadhikari et al. 2025). Since CMA does not provide information about the orientation or structural context of copy number variants, our findings further support that OGM is a valuable tool to resolve the structure of such variants and to overcome the limitations of CMA. This complex rearrangement affects the genes *POLA1, ARX* and *IL1RAPL1*, all of which are linked to neurodevelopmental disorders (Van Esch et al. 2019)(Sherr 2003; Montani et al. 2019), suggesting that it may be causative for the patient´s phenotype. Interestingly, structural variants and rearrangements involving the *IL1RAPL1* locus have already been implicated in autism spectrum disorder and intellectual disability, resp. (Youngs et al. 2012; Laino et al. 2016). Furthermore, a recent study described a case in which *IL1RAPL1*-involving duplications initially identified by CMA were resolved by OGM as part of a complex chromosomal rearrangement. This rearrangement might disrupt regulatory elements for *IL1RAPL1* expression, thereby contributing to the clinical phenotype (Dharmadhikari et al. 2025). However, a limitation of the present study is that it was not possible to test other male maternal relatives, as it would have been valuable for determining whether this complex rearrangement is truly the pathogenic aberration responsible for the patient’s phenotype. (Pei et al. 2025) recently suggested that, for linked interspersed duplications, only molecules completely spanning the duplicated regions or relevant junctions should be retained for analysis. In the present study, we performed a standard clinical OGM analysis, including molecule-level inspection, but did not systematically apply this approach. It can be considered in future analyses of comparable complex duplication events. Taken together, these two cases illustrate the general strength of OGM in detecting complex rearrangements that would have remained undetected by standard diagnostic methods. This aligns with a recent study in which OGM revealed that a translocation initially identified by conventional cytogenetic analysis in a patient with a neurodevelopmental disorder was in fact a complex rearrangement involving multiple breakpoints (Xiao et al. 2024).

In patient 7, OGM was helpful for confirmation and better characterization of a small deletion of the *SCN9A* gene, recognized by short-read exome sequencing, in a patient suffering from epilepsy. While CMA could not reliably detect the deletion, OGM successfully confirmed it in the index patient, but also in the unaffected father, suggesting that this SV is rather not causative for the patient’s epilepsy. Variants in *SCN9A* are held responsible for congenital insensitivity to pain and neuropathy, among others, while its role in the pathogenesis of epilepsy is controversial (Drenth and Waxman 2007; Fasham et al. 2020). In our patient, OGM technically helped to reliably exclude relevance of the discovered *SCN9A* variant.

However, is important to be aware of the limitations of SV-based analyses. For example, in this study several additional SVs were identified and reported in the results section, affecting genes potentially relevant to the patients' phenotypes. Interpreting the pathogenicity of these SVs, however, remains challenging due to the lack of comprehensive data in SV databases. While resources such as gnomAD-SV and DECIPHER exist, they still lack sufficient data coverage to allow reliable classification. In the future, the expansion of SV databases may improve structural variant classification and facilitate clinical OGM-based diagnostics.

Besides patients with known (mostly cytogenetic) aberrations, we also included three unsolved cases with strong clinical suspicion of hereditary neuropathy, since recent reports suggested that this approach could potentially detect causative SVs that are often missed with WES. For example, a heterozygous 85 kb microdeletion on chromosome 4p was identified with OGM and long-read sequencing as causative for an autosomal dominant form of CMT in a family with multiple affected individuals (Hsueh et al. 2023). Further, OGM recently revealed an intrachromosomal insertion from 7q31.1 into Xq27.1 as novel mechanism underlying the very rare X-linked recessive CMTX3 (Rahikkala et al. 2024). The authors even suggested that genomic rearrangement analysis of Xq27.1 should be included in standard diagnostic pipelines for childhood-onset CMT. However, for the three neuropathy cases investigated in the present study, OGM did not identify any causative SVs or CNVs related to the phenotype. Even though the number of analyzed patients is far too small to draw any substantial conclusions, our data suggest that OGM alone may not be sufficient to solve a high proportion of unsolved CMT cases. Recent studies have shown that applying WGS, as compared to traditional genetic testing methods, may provide an additional diagnostic yield of approximately 19% (Kim et al. 2023; Record et al. 2024). Therefore, additional approaches including WGS and perhaps RNAseq will be applied for these patients in the near future.

In conclusion, while OGM did not yield new insights for hereditary neuropathy, the method proved beneficial to better characterize known, mostly cytogenetic, aberrations and in two cases revealed much more complicated aberrations as suspected from previous analyses, thereby enabling more accurate genetic diagnoses. Limitations remain, especially in the detection of ring chromosomes and in visualizing regions such as acrocentric, telomeric and centromeric areas, which are often masked or not labeled. However, the next-generation telomere-to-telomere reference genome (T2T-CHM13) has the potential to address these limitations and could enhance structural variant detection, particularly in pericentromeric and subtelomeric regions (Banu et al. 2024). Based on our findings, OGM may be particularly useful as a complementary method after routine diagnostics, especially when structural variants remain incompletely characterized. This applies especially to apparently simple aberrations that may in fact represent more complex rearrangements, as well as to ring chromosomes, where OGM can reveal additional rearrangements at the ring fusion site. Careful manual inspection and adjustment of filter settings are essential, particularly for rearrangements involving acrocentric chromosomes, terminal regions, and centromeric regions. Unraveling the genetic cause of a rare disorder can have far-reaching consequences for the affected individuals and their families, who associate such diagnosis with significant personal utility, extending beyond health-related aspects. More specifically, it provides the opportunity to improve self-knowledge, build social resources and make reproductive decisions as well as pave the way for individualized therapies (Kohler et al. 2017). Given that the information from a genetic diagnosis can have such a broad impact, it is essential to integrate and evaluate new technologies like OGM into genetic testing to ensure accurate diagnoses (Kohler et al. 2017; Pollard et al. 2021; Bogaert et al. 2024). In this context OGM could be considered as a complementary method to standard genetic testing, with the potential to advance our understanding of neurogenetic disorders.

## Supplementary Information

Below is the link to the electronic supplementary material.

Supplementary Material 1

## Data Availability

The data generated during and/or analyzed during the current study are available from the corresponding author on reasonable request.
